# Retrospective Analysis of Task-Specific Effects on Brain Activity After Stroke: A Pilot Study

**DOI:** 10.3389/fnhum.2022.871239

**Published:** 2022-06-02

**Authors:** Marika Demers, Rini Varghese, Carolee Winstein

**Affiliations:** ^1^Motor Behavior and Neurorehabilitation Laboratory, Division of Biokinesiology and Physical Therapy, Herman Ostrow School of Dentistry, University of Southern California, Los Angeles, CA, United States; ^2^Department of Neurology, Keck School of Medicine, University of Southern California, Los Angeles, CA, United States

**Keywords:** neurorehabilitation, neural plasticity, upper extremity, stroke, motor cortex, magnetic resonance imaging

## Abstract

**Background:**

Evidence supports cortical reorganization in sensorimotor areas induced by constraint-induced movement therapy (CIMT). However, only a few studies examined the neural plastic changes as a function of task specificity. This retrospective analysis aims to evaluate the functional brain activation changes during a precision and a power grasp task in chronic stroke survivors who received 2-weeks of CIMT compared to a no-treatment control group.

**Methods:**

Fourteen chronic stroke survivors, randomized to CIMT (*n* = 8) or non-CIMT (*n* = 6), underwent functional MRI (fMRI) before and after a 2-week period. Two behavioral measures, the 6-item Wolf Motor Function Test (WMFT-6) and the Motor Activity Log (MAL), and fMRI brain scans were collected before and after a 2-week period. During scan runs, participants performed two different grasp tasks (precision, power). Pre to post changes in laterality index (LI) were compared by group and task for two predetermined motor regions of interest: dorsal premotor cortex (PMd) and primary motor cortex (MI).

**Results:**

In contrast to the control group, the CIMT group showed significant improvements in the WMFT-6. For the MAL, both groups showed a trend toward greater improvements from baseline. Two weeks of CIMT resulted in a relative increase in activity in a key region of the motor network, PMd of the lesioned hemisphere, under precision grasp task conditions compared to the non-treatment control group. No changes in LI were observed in MI for either task or group.

**Conclusion:**

These findings provide preliminary evidence for task-specific effects of CIMT in the promotion of recovery-supportive cortical reorganization in chronic stroke survivors.

## Introduction

Growing evidence suggests that rehabilitation interventions that harness motor practice can drive the brain’s restorative capacity. In a seminal study, Nudo et al. (1996) demonstrated evidence for cortical reorganization in the areas representing the distal forelimb in primates, not simply from spontaneous recovery, but as a result of motor skill training. Similar training-induced improvements in motor function and positive restorative neural plasticity in spared cortical regions have also been observed in other animal models (Castro-Alamancos and Borrell, 1995; Jones et al., 1999; Biernaskie and Corbett, 2001).

Intense task-specific motor training, such as Constraint-Induced Movement Therapy (CIMT), has been shown to reduce motor impairments in the paretic arm (Wolf et al., 2008; Corbetta et al., 2015; Kwakkel et al., 2015) and is thought to mediate sensorimotor recovery through experience-dependent cortical reorganization (Schaechter et al., 2002; Wittenberg et al., 2003; Liepert et al., 2006; Laible et al., 2012). Although CIMT, by definition, consists of a mitt constraint applied to the less-impaired arm, its most effective ingredient may be the *intensity* of practice (∼60 h over 2 weeks) of increasingly difficult tasks, known as “shaping,” combined with a transfer package for at-home practice (Winstein et al., 2003; Kaplon et al., 2007; Taub et al., 2013; Winstein and Wolf, 2014).

Intensive shaping practice can induce experience-dependent plasticity in the primary and secondary motor cortical regions of the lesioned hemisphere (Johansen-Berg et al., 2002; Wittenberg et al., 2003; Kwakkel et al., 2015). In the last decade, there is evidence that the behavioral improvements induced by CIMT are associated with cortical reorganization in sensorimotor areas (Levy et al., 2001; Johansen-Berg et al., 2002; Jang et al., 2003; Wittenberg et al., 2003; Kim et al., 2004; Schaechter and Perdue, 2008; Laible et al., 2012). For example, a study using transcranial magnetic stimulation showed increased motor map size in the primary motor cortex of the lesioned hemisphere in stroke survivors undergoing CIMT compared to a control group (Wittenberg et al., 2003).

One of the key tasks practiced during CIMT is the precision grasp, where the object is pinched between the flexor aspect of the fingers and that of the opposing thumb (Napier, 1956). Precision grasp involves finger individuation, and anticipatory movement planning to perform goal-directed, skilled actions. In contrast, power grasp involves undifferentiated finger and thumb movements of the whole hand for force control (Napier, 1956). Two cortical brain regions associated with precision and power tasks are the dorsal premotor cortex (PMd) and the primary motor cortex (MI), respectively. PMd is active during movement preparation, action selection and online control of reaching movements (Kantak et al., 2012). MI is associated with force control and movement execution (Cramer et al., 2002; Johansen-Berg et al., 2002). Our previous work demonstrated that a 2-week CIMT intervention resulted in improved anticipatory planning of hand posture selection, particularly in situations that require precision grasp actions, and improved movement time in reach-to-grasp tasks (Tan et al., 2012). We reason that dexterous and manipulative tasks practiced in the context of CIMT would more likely engage circuits involved in anticipatory planning than primarily force control or strength tasks (Muir and Lemon, 1983; Carey et al., 2005; Kantak et al., 2012). Considering typical inter-hemispheric competition from the contralesional hemisphere (Bütefisch et al., 2008), the restraint of the less-affected hand with intensive task-shaping of the more-affected hand may further promote recovery-supportive plasticity by driving activation back to the ipsilesional hemisphere in both MI and PMd.

Functional brain imaging studies of stroke motor recovery have primarily used undifferentiated finger tasks. Only a few studies have examined neural plastic changes as a function of task specificity and motor learning (Carey et al., 2005; Schaechter and Perdue, 2008). This provoked us to reanalyze an unpublished dataset from a companion study of the EXCITE trial (Winstein et al., 2003) in pursuit of evidence for cortical reorganization induced through task-specific training in chronic stroke survivors. The EXCITE trial tested the effectiveness of a 2-week multisite CIMT program compared to usual care on arm and hand function among stroke survivors within the first-year post-stroke. We selected two different grasp tasks: a precision grasp task involving force modulation through differentiated finger movement and a power grasp task involving force modulation through undifferentiated finger movement. A comparison of the neural activation pattern elicited from these two fundamentally different grasp tasks would allow a direct examination of the task-specific effects of CIMT and provide evidence about how task-specific training might modulate recovery-supportive functional plasticity.

This proof-of-concept analysis aims to evaluate the functional brain activation changes during a precision and a power grasp task in chronic stroke survivors who received 2 weeks of CIMT compared to a no-treatment control group. We selected two motor regions of interest (ROIs), PMd and MI, for their significant involvement in motor network changes obtained from both preclinical animal model and human clinical reports of upper limb recovery after stroke (Nudo, 2007; Buma et al., 2010; Calautti et al., 2010). We hypothesize that the precision task, more than the power task, would elicit greater brain activation of PMd of the lesioned hemisphere in the CIMT compared to the non-CIMT group. We expect differential group effects of task for change in laterality index for both ROIs.

## Materials and Methods

### Participants

Fourteen chronic stroke survivors (5–12 months post-stroke) with mild-moderate motor impairments were randomized to CIMT (*n* = 8) or non-CIMT (*n* = 6). Participants with functional magnetic resonance imaging (fMRI) safety contraindications and severe cognitive impairments were excluded. Only participants with a nearly complete set of evaluable behavioral and fMRI for baseline and immediate post-intervention visits were included in this retrospective analysis. All participants signed an informed consent approved by the Health Sciences Institutional Review Board of the University of Southern California.

### Intervention

The CIMT group completed the signature CIMT protocol, which consisted of a mitt constraint applied to the less-impaired arm and intensive, supervised task-specific training 6 h/day, 5 days/week, for 2 weeks (Winstein et al., 2003). Specifically, the less-impaired arm was placed in a mitt constraint for 90% of waking hours. Participants also received shaping (adaptive practice of functional tasks) and standard task training of the paretic limb for 6 h per day. The non-CIMT group completed the behavioral testing and fMRI scans 2 weeks apart, but received no formal rehabilitation training.

### Behavioral Measures

Before and after a 2-week period, the 6-item Wolf Motor Function Test (WMFT-6) and the Motor Activity Log (MAL) were administered by a blinded assessor. The WMFT-6 is a subset of the WMFT, includes six time-based precision and dexterous tasks and is strongly correlated with the 15-item WMFT (*r* = 0.61, *p* = 0.02; Wolf et al., 2005). The MAL is a self-reported measure in which participants rate the quantity, Amount of Use (AOU) and Quality of Movement (QOM) of the paretic arm during 30 everyday activities performed over the past 3 days (Taub et al., 1993; Uswatte and Taub, 1999). Each activity is scored on a 6-point scale with higher scores indicating better performance. The upper limb Fugl-Meyer motor assessment (FMA; Fugl-Meyer et al., 1975) was also administered at baseline to determine impairment level (0: severe motor impairments to 66: normal function).

### Functional MRI Task Description

**FIGURE 1:**
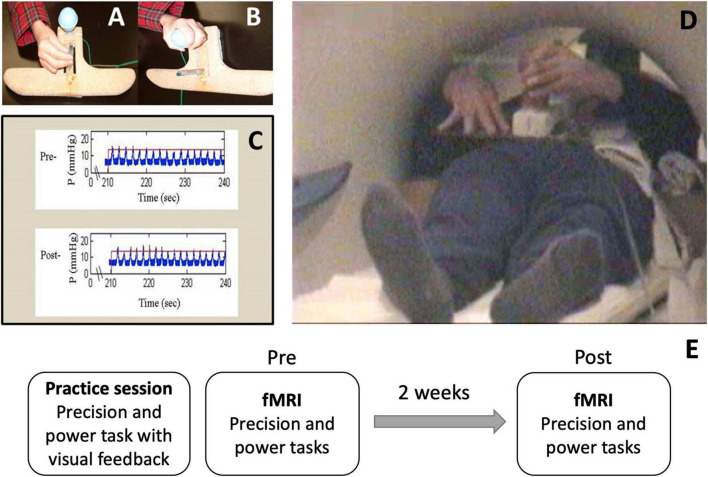
Motor Activation Tasks performed in the functional magnetic resonance imaging (fMRI) scanner. Task apparatus MRI safe device with two pneumatic pressure sensors for **(A)** precision grip and **(B)** power grasp. **(C)** Sample pre- and post-pressure and rate graphs demonstrating participant ability to maintain 50% of their predetermined maximum pressure and 75% of their predetermined maximum rate throughout each fMRI session. **(D)** Participants were positioned to minimize movement within the scanner. No visual feedback was offered during the scan. **(E)** Study timeline.

Chronic stroke survivors underwent functional MRI using a block design before and after a 2-week period. Each fMRI session included four 30-s task blocks alternated with five 30-s rest blocks (4.5-min run time and 108 volumes collected per run) for each grasp (precision, power) and for each limb (affected, less-affected), totaling four runs per fMRI session. Only the results for the affected limb are presented here. Participants repetitively compressed either a vertically mounted plastic tube with the index and middle fingers against the thumb in a precision grasp posture (precision grasp), or a vertically mounted rubber bulb with all digits in a power grasp posture (power grasp) (Figures 1A,B,D). A custom-built fMRI-compatible apparatus with the two grasp units was connected to a pneumatic pressure transducer. The output from the pressure transducers were collected electronically using custom MATLAB software for subsequent off-line analysis (dataWizard, v. 0.9)^1^. Prior to each fMRI session, participants practiced each grasp task with the paretic hand, in the supine position, outside of the scanner to: (1) establish and ensure across-session consistency of pressure level and rate; (2) reduce mirror movements of the opposite arm and associated head, elbow and shoulder movements. During the practice period, participants were offered visual feedback about pressure production to maintain a consistent pre-specified pressure and rate (Figure 1C). Once a participant was able to perform each grasp task with a consistent pressure and rate without feedback, they were scheduled for the fMRI sessions (Figure 1E). During the scan, participants were reminded to maintain the same pressure and rate that they had practiced, i.e., 50% of their maximum pressure and 75% of their maximum rate, without feedback. These levels were chosen to (1) account for a range of individual differences in force capability and motor control and (2) avoid fatigue during the fMRI scanning blocks. Since our participants had mild-moderate motor impairments and the pressure levels were submaximal, the estimated force levels used during the scan runs were likely closer to functional levels. The goal for the second scan was based on the baseline rate and pressure levels used during the first scan to maintain consistent pressure levels across the two runs (pre and post).

### Functional MRI Data Acquisition

Functional and structural images were acquired using a 1.5 Tesla Siemens Sonata scanner at the Brain Mapping Center, University of California, at Los Angeles. Auto-shimming was conducted at the beginning of the scan to correct magnetic inhomogeneity. All functional and structural images covering the whole brain were acquired parallel to the anterior-posterior commissure line using a sagittal localizer. Three dimensional (3D) high resolution T1-weighted images were acquired for anatomical localization [repetition time (TR) = 1,970 ms, echo time (TE) = 4.38 s, flip angle = 15°, voxel size = 1 × 1 × 1 mm; matrix = 256 × 256]. A set of two dimensional T1-weighted inversion time echo-planar images consisting of 25 contiguous slices was acquired before each functional run (TR = 600 ms; TE = 15 ms; flip angle = 90°, matrix = 128 × 256; in-plane resolution of 1.5 × 0.8 × 4 mm with 1 mm gap). For functional scans, T2*-weighted echo-planar image with BOLD contrast (Kwong et al., 1992; Ogawa et al., 1992) were acquired (TR = 2,500 ms, TE = 60 ms, flip angle = 80°; matrix = 64 × 64; voxel size = 3 × 3 × 4 mm with 1 mm gap). A total of 108 volumes were acquired for each functional run.

### Data Analysis

Baseline demographic and clinical data were compared between groups using independent *t*-tests or χ^2^ analyses. fMRI data were analyzed using the FSL software (FMRIB Software Library 3.1). Image preprocessing steps included: (1) spatial realignment to the center volume for motion correction, (2) co-registration of functional images with the high-resolution structural scan using a seven-parameter rigid body transformation, and (3) spatial smoothing using a five-mm full width-half-maximum Gaussian kernel. Two *a priori* ROIs associated with neuroplastic changes after stroke [bilateral MI and PMd cortices] were selected. MI is defined as the gyrus between central sulcus and precentral sulcus including the hand knob. PMd includes the gyrus dorsal from the precentral sulcus not exceeding 10 mm. Cluster-based activation Z-maps were constructed to calculate the mean number of activated voxels with a threshold of Z > 3.1 (corresponding to a *P*-value < 0.01, corrected for multiple comparisons) in both ROIs. There was lesion overlap with the M1 ROI for two participants, one in the CIMT group and one in the non-CIMT group, but not for the PMd ROI.

Voxel counts were computed and used to calculate a laterality index for each ROI [LI = (C-I)/C + I)]; where C and I indicate contralateral (lesioned) and ipsilateral (non-lesioned) activation to the grasping hand, respectively. LI ranges from 1 (activation only of the ROI of the lesioned hemisphere) to −1 (activation only of the ROI of the non-lesioned hemisphere). LI change scores (post-pre) were calculated for each ROI, in each group, for each grasp condition. If the voxel count number for both hemispheres were zero for a given participant, LI was not calculated for that condition.

### Statistical Analysis

Analyses were conducted using the R statistical computing package (version 3.5.1). Within-group behavioral data were compared using the Wilcoxon signed rank test (two-tailed). A multiple linear regression was performed to estimate the task-specific effects of the intervention on ΔLI in two separate models (PMd and MI). To test the hypothesis that the precision task, more than the power, would elicit greater activation of the lesioned PMd/MI in the CIMT compared to the non-CIMT control group, we included an interaction term (group x task) and performed *post-hoc t*-test comparison to determine the locus of the interaction. Standard errors and 95% confidence intervals (CI) for regression estimates were confirmed over 1,000 bootstrap replicates.

Using a backward selection approach, we included potential confounding variables—age, sex, chronicity, lesion volume, lesion side and FMA—one at a time and preserved any variable that met a liberal cut-off of *p* = 0.2. Continuous variables were assessed for normality using QQ plots and Shapiro-Wilk tests. Of these, the distribution for lesion volume was extremely positively skewed and was log-transformed. None of the potential confounding variables met the significance cut-off criterion and were therefore not included in the final model. All necessary assumptions for generalized linear models were tested when appropriate.

## Results

No statistically significant differences were found between groups for any demographic or stroke characteristics at baseline (Table 1), except for the MAL QOM which was lower for the non-CIMT group. LI was not computed for two participants in the CIMT group (one for MI and one for MI and PMd during the power task) and one in the non-CIMT group (PMd during the power task) due to a voxel count of zero in at least one hemisphere. Lesion size and location varied in both groups (Supplementary Figure 1). Limb concordance (i.e., dominant hand is more affected) was reported in 3/8 CIMT and 2/6 non-CIMT participants.

**TABLE 1:** Characteristics of the constraint-induced movement therapy (CIMT) group and non-CIMT group and effect of CIMT treatment on behavioral outcomes

Groups	ID	Age (y)	Sex	Dominant hand	Affected hand	Time from stroke onset (mo)	Initial Fugl-Meyer UE motor score (max = 66)	WMFT-6 (s)			MAL		
								Pre	Post	Pre-AOU	Post-AOU	Pre-QOM	Post-QOM
CIMT	01	57	M	R	L	10.5	47	6.21	4.53	2.20	2.11	3.92	4.21
	02	58	F	L	L	5.2	45	71.80*^2^*	17.09	1.38	3.68	2.29	3.28
	03	38	M	R	L	6.1	51	70.43*^3^*	52.71*^2^*	0.67	1.25	2.33	2.18
	04	57	M	R	R	8.1	50	49.66*^1^*	13.10	3.00	4.38	3.31	4.12
	05	63	M	R	L	5.8	57	22.50	11.18	2.55	3.35	3.55	3.60
	06	80	M	R	L	5.7	51	11.10	7.99	4.17	4.37	3.10	3.85
	07	73	M	R	L	7.0	53	6.03	4.36	3.58	4.05	3.44	3.91
	08	55	F	R	R	9.2	53	8.87	4.44	2.47	4.20	3.52	4.10
**Median/count (SEM)**	***N* = 8**	**57.5 (4.44)**	**2F/6M**	**7R/1L**	**2R/6L**	**6.5 (0.67)**	**51 (1.32)**	**16.80 (10.15)**	9.85* (5.71)	**2.51 (0.39)**	**3.87 (0.41)**	**3.38 (0.21)**	**3.88 (0.24)**
NON-CIMT	09	51	M	R	L	6.1	52	6.18	4.10	0.96	2.18	0.84	1.41
	10	26	F	R	R	11.8	63	7.54	5.34	1.71	2.90	3.23	4.09
	11	69	F	R	L	8.6	49	4.55	4.06	2.22	2.24	2.95	3.19
	12	76	F	R	R	8.4	37	2.05	1.91	1.41	3.10	1.36	1.62
	13	51	F	R	L	10.8	44	101.97*^5^*	118.5*^5^*	1.04	2.86	0.67	2.03
	14	68	M	R	L	11.4	51	36.01	14.60	0.74	0.79	1.17	1.21
**Median/count (SEM)**	***N* = 6**	**59.5 (7.44)**	**4F/2M**	**6R/0L**	**2R/4L**	**9.7 (0.89)**	**50 (3.55)**	**6.86 (15.96)**	**4.72 (18.84)**	**1.23 (0.22)**	**2.55 (0.35)**	**1.27 (0.45)**	**1.83 (0.47)**

CIMT, Constraint Induced Movement Therapy; MAL, Motor Activity Log, AOU, amount of use scale, QOM, quality of movement scale; SEM, standard error; UE, Upper extremity. WMFT-6, Six-item Wolf Motor Function test. Within-group comparison were done using the two-tailed Wilcoxon signed rank test. Median/count (standard error) are indicated in bold. Number of incomplete items (those not completed in 120s) in the WMFT-6 are indicated by an italic superscript above the mean time score. For the MAL-AOU and QOM, a score of 5 is the maximum score. An improvement in arm and hand performance is indicated by an increase in the mean MAL score and a decrease in the mean WMFT-6 time. The CIMT group demonstrated a significantly faster mean WMFT-6 time compared to the non-CIMT group. For the MAL, both groups showed a trend toward greater improvement, but when adjusted for multiple comparisons were not statistically significant. *Indicates significance (p < 0.017) for the within-group comparisons between baseline and post-assessment.

At the behavioral level, participants in the CIMT group showed significant improvements in the WMFT-6 (*p* = 0.008), but not the control group (*p* = 0.313). For the MAL, both groups showed a trend toward greater improvement (AOU CIMT: *p* = 0.016, non-CIMT: *p* = 0.031, QOM: CIMT: *p* = 0.023, non-CIMT: *p* = 0.031), but when adjusted for multiple comparisons were not statistically significant.

### Precision Task Elicits Greater Relative Activation of Premotor Cortex of the Lesioned Hemisphere in Constraint-Induced Movement Therapy Group Compared to Controls

Our final model for the PMd ROI was significantly different from a null model [*F*(4, 20) = 4.65, *p* = 0.012, adj. *R*^2^ = 32.3%]. However, that for the MI ROI was not significant [*F*(4, 20) = 1.05, *p* = 0.389, adj. *R*^2^ = 0.72%].

**FIGURE 2:**
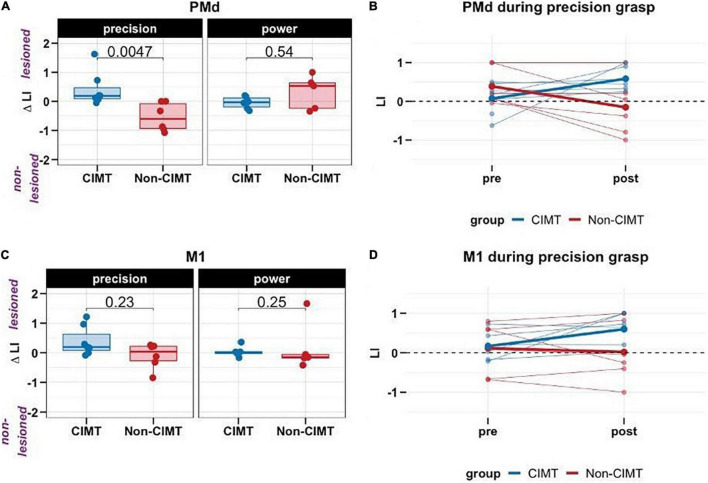
Change in laterality index (LI) across groups and tasks (positive values in ΔLI indicate greater activation of the ROI on the lesioned relative to non-lesioned hemisphere). **(A)** For the precision task, compared to the non-CIMT group (*n* = 6), the constraint-induced movement therapy (CIMT) group (*n* = 7) showed increased activation of the dorsal premotor cortex (PMd) in the lesioned hemisphere. However, this effect was not observed for the power task (CIMT group, *n* = 6, non-CIMT group, *n* = 5). **(B)** Individual changes in LI from pre-post in PMd; thicker lines are group means **(C)** In the primary motor cortex (MI), no changes in LI were observed from pre to post for either group. **(D)** Individual changes in LI from pre-post in MI; thicker lines are group means.

Consistent with our hypothesis, there was a significant interaction between group and task (*B* = 1.31, *p* = 0.004). The *post hoc* analysis confirmed the locus of the interaction; specifically for the precision task, the CIMT group showed an increase PMd ΔLI, i.e., increased activation of the PMd of the lesioned hemisphere relative to the non-lesioned hemisphere, compared to the non-CIMT group (*t* = 3.458, *p* = 0.002; Figure 2A). This group level change was apparent on an individual level (Figure 2B); PMd ΔLI increased toward the lesioned hemisphere in 6/7 participants for the CIMT group, while LI decreased in 5/6 participants for the non-CIMT group. See Supplementary Figure 2 for fMRI activation maps from two representative participants (CIMT group ID 02, non-CIMT group ID 11) for each task and each time point.

There was also a significant main effect of “*task”* (Table 2) such that compared to the power task, PMd ΔLI was smaller for the precision task, when averaged between the two groups. Change in activation of PMd tended to be smaller for the precision compared to the power grasp. However, given the strong interaction between group and task (i.e., the strong effect of group on PMd ΔLI for the precision but not power task), interpreting this main effect on its own, by averaging between groups, is misleading.

**TABLE 2:** Estimates from multiple linear regression.

	PMd Δ LI			MI Δ LI		
Predictors	Estimates	CI	*p*	Estimates	CI	*p*
(Intercept)	0.32	−0.14 to 0.78	0.166	0.17	−0.32 to 0.66	0.473
Group	−0.36	−0.98 to 0.26	0.241	−0.13	−0.79 to 0.52	0.675
Task	−0.86	−1.48 to −0.23	**0.010**	−0.27	−0.93 to 0.39	0.402
Group × task	1.31	0.46−2.16	**0.004**	0.63	−0.27 to 1.52	0.159
Observations	24			24		
*R*^2^/Adjusted *R*^2^	0.411/0.323			0.137/0.007		

*Significant p-values are denoted in bold.*

As noted earlier, for the MI ROI, there was no significant difference between CIMT and non-CIMT for either task (Figures 2C,D).

## Discussion

In this retrospective analysis, we found that compared to a no-treatment control group, 2 weeks of CIMT resulted in a relative increase in activity in a key node of the motor network, the PMd of the lesioned hemisphere under precision grasp task conditions. The results underscore the importance of task-specific training in the context of CIMT and its potential for driving motor network activity toward restoration by increasing paretic arm function and hand use, as supported by improvements in the WMFT-6 and a trend toward improved ratings on both scales of the MAL. However, contrary to our hypothesis, no significant changes in MI brain activation were observed for the precision or power tasks in either group. These findings are preliminary in view of the small sample size, stressing the need to be replicated in a larger and similarly rigorous study.

The PMd ΔLI findings corroborate the effect of CIMT in driving neuroplasticity. These results were complimented by behavioral changes (though not significant for the MAL) both in precision task performance (WMFT-6) and perceived quantity and quality of arm and hand activities (MAL) in the CIMT group. Previous studies report evidence for cortical reorganization in sensorimotor areas induced by task-specific training (Levy et al., 2001; Johansen-Berg et al., 2002; Jang et al., 2003; Kim et al., 2004), but do not differentiate between key neural nodes within the sensorimotor network associated with specific task conditions. Our results are consistent with previous work that emphasizes the role of PMd in recovery mechanisms and the precise regulation of force (Johansen-Berg et al., 2002; Ward et al., 2007; Bestmann et al., 2010). Consistent with the results from Bestmann et al. (2010), input from upstream areas such as PMd of the lesioned hemisphere might assist downstream brain regions to produce movement. Evidence supports the role of PMd in movement execution, especially for stroke survivors with greater motor impairments (Ward et al., 2007), and in the control of skilled movement beyond simple execution, that which involves strategy-based learning mechanisms (Kantak et al., 2012; Stewart et al., 2016; Spampinato and Celnik, 2021). One interpretation is that CIMT because of its repetitive and progressively more challenging practice, elicits motor learning and decision-making processes, including anticipatory action planning, which uniquely engages PMd in the selection of action. In our previous work (Tan et al., 2012), we provided evidence that the CIMT group demonstrated more optimal anticipatory hand posturing prior to precision grasp tasks than did the control group. These findings combined with our behavioral results here, complement the current fMRI findings and support the differential effect of CIMT on PMd. Our results also provide partial support for the hypothesis that the recovery of hand motor function following a stroke is mediated by separate systems for strength and dexterity (Xu et al., 2017; Mawase et al., 2020).

One aspect of our PMd laterality index change findings that surprised us was the apparent *decrease* in LI, toward the non-lesioned hemisphere that we observed for the no-treatment control group (red line in Figure 2B). One possible interpretation based on our behavioral results is that the mere exposure to the MAL questionnaire at baseline primes the individual to attempt more use of the paretic limb in everyday activities, especially for bimanual tasks as a support or stabilizer, but not necessarily for dexterous activities that would be emphasized as part of CIMT. If this was the case, that increased use could have manifested as compensatory activation of the non-lesioned hemisphere PMd and as increased perceived use and quality of use of the paretic limb that we noted for MAL amount and quality scores (Uswatte et al., 2006; Narai et al., 2016; Table 1). The fact that we did not see significant control group improvements for dexterous performance of the WMFT-6 is consistent with this explanation. Another explanation is that participants habituated to scanner environment and thus showed less brain activation at the post-intervention scan, however, this is unlikely because such habituation would be evident for both groups. Down-regulation of brain activity could also have been influenced by time since stroke, though this explanation is weak given that the non-CIMT group was only ∼3 months longer from stroke onset than the CIMT group (Lindberg et al., 2007).

This study adds to the literature by demonstrating that cortical reorganization associated with CIMT is a function of the specific tasks practiced and can be restorative rather than compensatory in nature (Hodics et al., 2006; Levin et al., 2009). The novelty of our study is that neural plastic changes were examined as a function of task type, unlike most studies that have focused primarily on whole hand grasp tasks.

Unlike PMd, we did not observe significant MI ΔLI for either group or task. We suggest the following interpretation for these null findings. The pressure level was kept constant over repeated fMRI sessions to control for the well-known relationship between force (i.e., muscle torque) and MI activation, and for performance (i.e., strength) gains that were expected to occur in the CIMT group but not necessarily in the non-CIMT group (Cramer et al., 2002). Our careful control to obtain consistent pressure and rate levels between scans may have biased the results toward observing differences in motor planning rather than execution. Another potential confounding factor is that two participants, one in the CIMT group and one in the non-CIMT group, had brain lesions directly affecting MI, but none had lesions directly affecting PMd. Further, those subjects for which we could not compute LI were from both groups, but only pertained to the power task. The task-specific nature of CIMT training may induce important changes upstream from M1 in motor areas responsible for higher-level task planning, including movement preparation and action selection, those functions for which PMd has an important role (Grafton et al., 1998; Hoshi and Tanji, 2007; Kantak et al., 2012). Evidence supports that the strengthened functional connectivity between PMd and MI in the ipsilesional hemisphere is associated with improved long-term retention of motor skills (Lefebvre et al., 2015, 2017). Taken together, the choice of MI for one of two pre-specified ROIs was perhaps naïve given that CIMT does not directly target hand strength and importantly, we constrained the pressure and rate levels to maintain consistent performance across fMRI sessions.

No study is without limitations. The fMRI data were collected using a 1.5 Tesla scanner, which produced lower image quality than what is now available for research. Given the high variability in the lesion locations and clinical presentation of stroke, the main limitation of this study is its small sample size. The lack of sufficient samples combined with the large variability across participants limits generalizability of our findings. Future studies should examine the relationship between the effects demonstrated here and lesion size and location. With a larger sample size, researchers could also pursue the search for hemispheric differences on precision and force control (Mani et al., 2013). It is possible that performance variability across participants during imaging added noise to the data that overwhelmed the signal. However, implementation of a rigorous pre-training phase outside of the scanner and individual-participant task performance criteria (% max pressure and rate) promoted performance consistency across scanning sessions and reduced the likelihood that activation was due to performance variability. On the other hand, this rigorous pre-training may explain the null effects observed for MI compared with those for PMd (i.e., differences in motor planning rather than execution). Finally, it is impossible to identify which elements of the CIMT intervention (i.e., nature of the tasks practiced, high treatment intensity, mitt constraint) drove cortical reorganization in the CIMT group. Further studies are required to elucidate the relationship between cortical reorganization and effective ingredients of CIMT.

Intense task-specific training of the affected limb in combination with restraint of the less-affected limb drives functional reorganization by shifting bi-hemispheric motor cortical activity toward the lesioned hemisphere. These results provide preliminary evidence from humans undergoing rehabilitation for the principle of specificity and intensity of experience-dependent plasticity derived primarily from animal models in the research laboratory (Kleim and Jones, 2008). These findings should be replicated in a larger study.

## Data Availability Statement

The original contributions presented in the study are publicly available. This data can be found here: https://osf.io/89mkd/.

## Ethics Statement

The studies involving human participants were reviewed and approved by the Health Sciences Institutional Review Board of the University of Southern California. The patients/participants provided their written informed consent to participate in this study.

## Author Contributions

MD: writing – original draft preparation and visualization. RV: formal analysis, software, data curation, visualization, and writing – reviewing and editing. CW: conceptualization, supervision, and writing – reviewing and editing. All authors contributed to the article and approved the submitted version.

## Conflict of Interest

The authors declare that the research was conducted in the absence of any commercial or financial relationships that could be construed as a potential conflict of interest.

## Publisher’s Note

All claims expressed in this article are solely those of the authors and do not necessarily represent those of their affiliated organizations, or those of the publisher, the editors and the reviewers. Any product that may be evaluated in this article, or claim that may be made by its manufacturer, is not guaranteed or endorsed by the publisher.

## Footnotes

1
https://sites.google.com/site/ucladatawizard/
