# Supplementary material for: Retrospective Analysis of Task-Specific Effects on Brain Activity After Stroke: A Pilot Study
Source: Front Hum Neurosci. 2022 Jun 2;16:871239. doi: 10.3389/fnhum.2022.871239 (PMC9201099; doi:10.3389/fnhum.2022.871239)
Supplement: Supplementary file 1 [file Data_Sheet_1.pdf]

Supplementary Material

1 Supplementary Figure 1. Lesion description

	ID	3D Image	3D Volume (cc) Location	ID	3D Image	3D Volume (cc) Location
CIMT	01	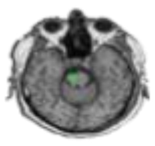	0.2 R Pontine	05	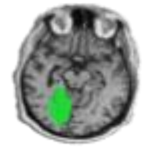	14.8 R O
	02	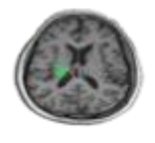	0.7 R IC	06	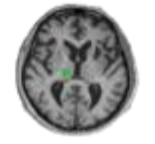	1.2 R IC
	03	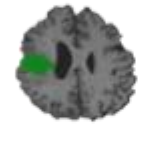	14.5 R M1	07	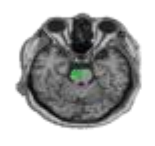	0.8 R Pontine
	04	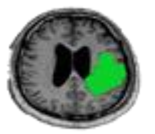	72.6 R P,T	08	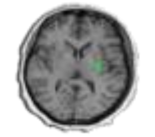	0.9 L IC
Non-CIMT	09	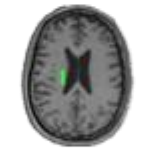	0.7 R CN,CR	12	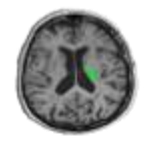	1.4 L CN,CR
	10	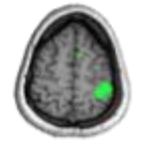	3.1 L M1	13	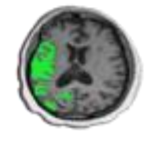	27.7 R SM,T
	11	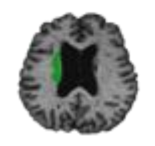	2.3 2R CN, CR	14	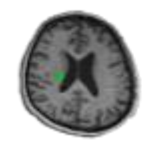	0.8 R CR,CN

Supplementary Figure 1. Legend: CIMT, constraint-induced movement therapy; R, Right; L, Left; B, brainstem; F, Frontal area; T, Temporal area; P, Parietal area; O, Occipital area; M1, primary motor area; SM, Sensorimotor area; CR, Corona radiata; CN, Caudate nucleus; IC, Internal capsule. *The green area in the 3D image shows the lesion on the slice with the largest computed area.

2 Supplementary Figure 2. fMRI activation maps during each task

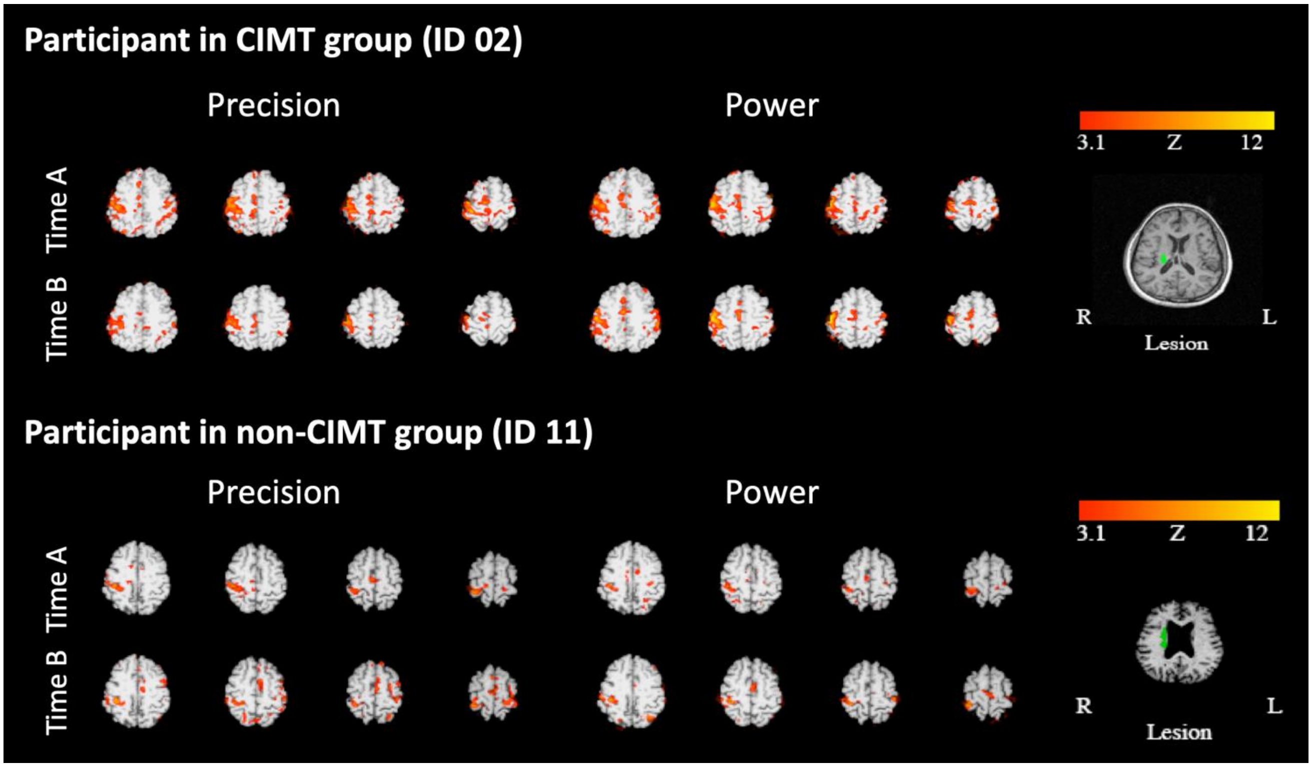

Supplementary Figure 2. Legend: fMRI activation maps during the precision and power grasp tasks. *Top*: The participant received constraint-induced movement therapy (CIMT) between Time A (pre-intervention) and B (post-intervention). *Bottom*: The participant on the bottom did not receive CIMT between Time A (pre) and B (after 2 weeks). For both participants, the affected limb is left. *Right panel*: The lesion is indicated in green on the slice with the maximum lesion volume. The Z frequency indicates the strength of the bold signal relative to rest, with greater bold signal in yellow.
